# Tracheoesophageal Injury Following Gunshot Wound to the Chest: A Case Report

**DOI:** 10.7759/cureus.100083

**Published:** 2025-12-25

**Authors:** Eliseo A. Gonzalez-Sanchez, Dario E. Medina-Muñoz, Manuel F. Bermea-Caldelas, Marco A Hernández Guedea, Gerardo E Muñoz-Maldonado

**Affiliations:** 1 General Surgery, Dr. José Eleuterio Gonzalez University Hospital, Autonomous University of Nuevo León, Monterrey, MEX

**Keywords:** dual surgical approach, esophageal perforation, gunshot wound, thoracic trauma, tracheal injury, tracheoplasty

## Abstract

Thoracic tracheal injuries resulting from gunshot wounds are extremely rare traumatic events associated with high mortality rates, necessitating immediate airway and surgical intervention. We present the case of a 46-year-old man with a gunshot wound to the chest that resulted in an 80% thoracic tracheal laceration coupled with an associated esophageal perforation. The patient presented with severe dyspnea, extensive emphysema, and ineffective ventilatory mechanics despite initial management. Emergency surgical management involved a median sternotomy for tracheal exposure and repair. However, due to an intraoperative cardiac arrest, an emergent left anterior thoracotomy was required for direct cardiac massage and subsequent esophageal management. A primary terminal-to-terminal tracheoplasty was performed, and the esophageal injury was repaired using an intercostal muscle patch. The patient was extubated uneventfully on postoperative Day 3 and was discharged 10 days later. This case highlights the need for rapid and decisive action in complex tracheal injuries. The combined dual surgical approach (sternotomy and emergency thoracotomy) proved to be a viable and potentially life-saving strategy in the context of critical penetrating chest trauma.

## Introduction

Tracheobronchial injuries resulting from penetrating thoracic trauma, such as gunshot wounds, are clinical entities of extremely low incidence, occurring in less than 1% of chest trauma cases, yet they represent one of the most critical surgical emergencies [1,2]. The clinical gravity of these injuries is significantly compounded when they are associated with esophageal perforations, a combination that drastically increases mortality rates due to the risk of fulminant mediastinitis and the potential development of tracheoesophageal fistulas (TEFs) [3]. Managing such complex injuries requires not only rapid airway securement but also a strategic surgical approach to address multi-organ damage in hemodynamically unstable patients [2].

The selection of the surgical incision is a decisive factor in trauma outcomes and is typically dictated by the anatomical location of the tracheal lesion [1,4]. While a median sternotomy is considered the standard approach for providing optimal exposure to the upper and middle thoracic trachea, its utility may be limited in the face of sudden physiological collapse [4,5]. In scenarios involving intraoperative cardiac arrest or the need for direct cardiac massage and access to posterior mediastinal structures, the literature supports the immediate transition to or addition of an emergent left anterior thoracotomy [4,5]. This dual surgical strategy, though aggressive and non-standard, serves as a vital resuscitative maneuver to re-establish spontaneous circulation while simultaneously managing injuries to the esophagus or descending aorta [5].

Definitive repair in these cases often necessitates primary end-to-end tracheoplasty and meticulous esophageal reconstruction [1,6]. A critical technical element to prevent postoperative morbidity is the interposition of viable, vascularized tissue, such as a pedicled intercostal muscle patch, between the suture lines to preclude fistula formation [3]. Given the rarity of combined tracheoesophageal trauma, standardized guidelines remain limited, making the documentation of successful, high-complexity interventions essential for the surgical literature [2,6].

## Case presentation

**Figure 1:**
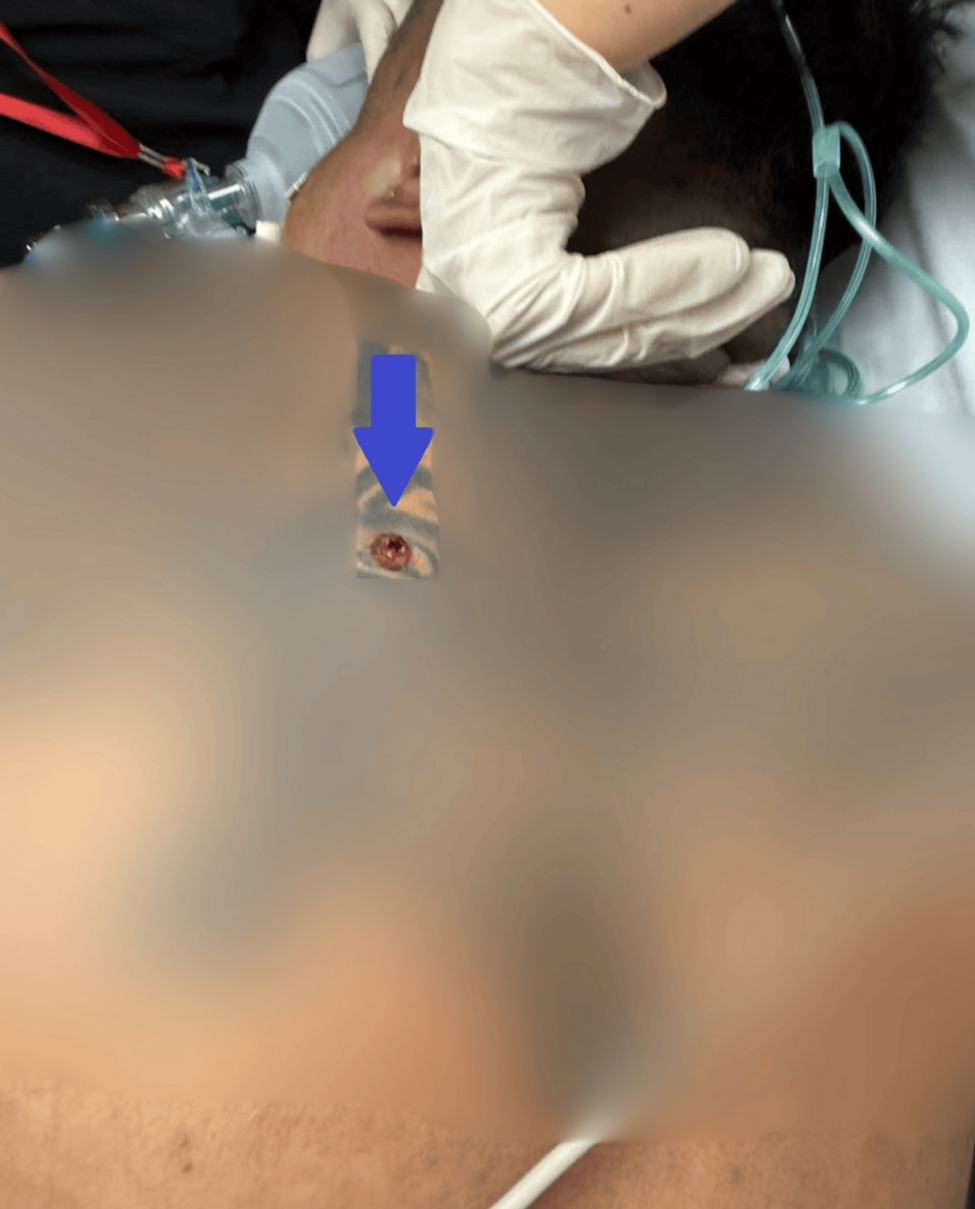
Posterior view of the back showing the entry wound (indicated by the blue arrow) from a firearm projectile, located in the left paravertebral region at approximately the level of T3.

**Figure 2:**
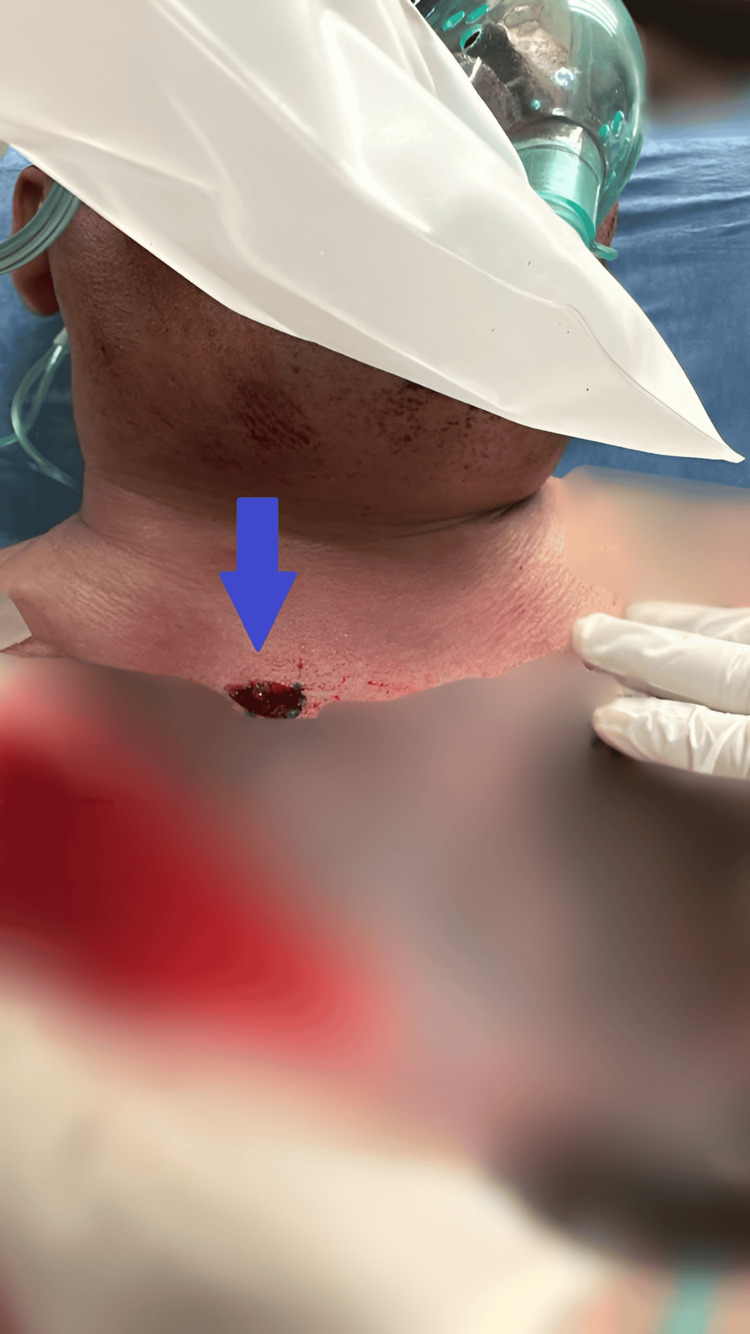
Anterior view of the neck showing the exit wound (indicated by the blue arrow) from a firearm projectile, located approximately 2 cm below the middle third of the right clavicle.

**Figure 3:**
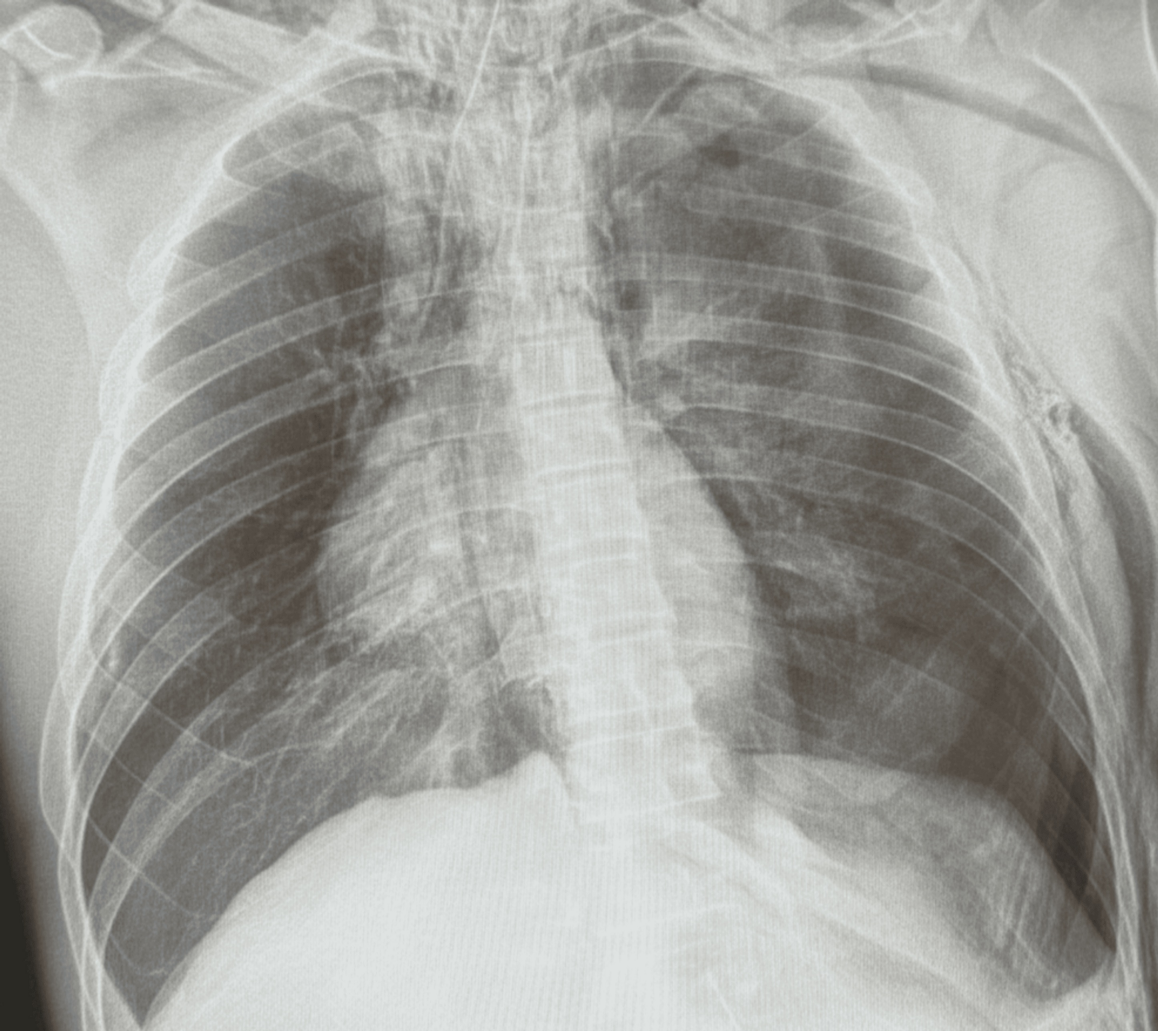
Anteroposterior chest radiograph obtained in the resuscitation room upon the patient’s arrival, demonstrating a left pneumothorax, subcutaneous emphysema, pneumomediastinum, and pneumopericardium.

We present an otherwise healthy 46-year-old man who sustained an entry gunshot wound to the interscapular region of the posterior chest (Figure 1) and an exit wound to the anterior chest in the right supraclavicular area (Figure 2). Upon arrival at the emergency department, the patient was unconscious with severe hemodynamic instability, presenting a blood pressure of 80/40 mmHg, a heart rate of 125 bpm, and a respiratory rate of 32 breaths per minute. Extensive subcutaneous emphysema and severe dyspnea were noted. Immediate endotracheal intubation was performed via conventional direct laryngoscopy due to the critical nature of the airway compromise. Initial chest radiography demonstrated a left pneumothorax, subcutaneous emphysema, pneumomediastinum, and pneumopericardium (Figure 3).

An orotracheal tube was initially placed; however, due to inadequate ventilation, a new endotracheal tube was inserted directly into the distal segment of the transected trachea during the median sternotomy (Video 1). The surgery consisted of a median sternotomy with exposure of the thoracic trachea, which revealed an 80% laceration with the endotracheal tube displaced toward the mediastinum. Emergent airway management was performed, and tracheal intubation was secured 3 cm above the carina.

**Video 1:** Intraoperative video obtained through a median sternotomy showing the orotracheal tube placed in the distal segment of the trachea to secure the airway.

The choice of surgical incision is critical and often pre-determined by the location of the tracheal injury. While a median sternotomy provides excellent exposure to the upper and middle thoracic trachea, the unexpected complication of cardiac arrest mid-procedure necessitated an immediate shift in strategy. The patient presented a loss of pulse, and an emergent left anterior thoracotomy was performed. Literature supports the use of thoracotomy for immediate reanimation and to address severe trauma to posterior mediastinal structures (like the esophagus and descending aorta) or pulmonary hilum, particularly when initial access is insufficient or the patient codes. Upon four minutes of direct cardiac massage and optimized ventilation, the patient presented a spontaneous circulation response. The successful re-establishment of circulation, followed by the definitive repair, underscores that this combined surgical approach, although unconventional, is a valid and life-saving strategy in critical, unstable penetrating chest trauma where an initial approach proves insufficient.

Definitive management involved an end-to-end tracheoplasty to restore airway continuity and integrity using 4-0 monofilament absorbable interrupted sutures (PDS). Crucially, the associated esophageal injury (approximately 30% of the circumference) was repaired in two layers: the mucosa with 4-0 absorbable sutures and the muscular layer with 4-0 silk interrupted sutures. The anastomosis was protected using a pedicled intercostal muscle patch placed between the tracheal and esophageal repairs. This technique is recognized in the literature as essential for preventing a disastrous TEF, which is a common and morbid complication of combined injuries in this region.

Bilateral endopleural tubes and a mediastinal drain were placed. The patient’s rapid and uneventful recovery, with extubation on day three and discharge on day 10, validated the effectiveness of the rapid diagnosis, immediate securement of the airway, and the robust, multilayered repair strategy. Follow-up via bronchoscopy and a water-soluble contrast test confirmed the integrity of the anastomosis without evidence of leakage. The procedure was performed at the Dr. José Eleuterio González University Hospital, Monterrey, Mexico.

The clinical timeline of key interventions and outcomes is summarized in Table 1.

**Table 1:** Clinical timeline of key interventions and outcomes.

Key Event	Findings/Intervention	Time Since Admission
Emergency Room Admission	Gunshot wound to the chest. Severe dyspnea, extensive emphysema, unconsciousness.	T0 (Arrival)
Initial Stabilization	Intubation and resuscitation efforts. Left chest tube insertion.	T0 + 10 minutes
Emergency Surgery (Phase 1)	Start of median sternotomy due to ineffective ventilatory mechanics. Finding: 80% thoracic tracheal laceration.	T0 + 30 minutes
Critical Complication	Cardiac arrest (loss of pulse). Emergent left anterior thoracotomy for direct cardiac massage.	Intraoperative
Definitive Repair (Phase 2)	End-to-end tracheoplasty. Esophageal repair protected by an intercostal muscle patch.	Intraoperative
Follow-up	Successful extubation after clinical stability. Bronchoscopy and water-soluble test confirmed integrity.	Postoperative Day 3
Hospital Discharge	Patient discharged to continue follow-up at the outpatient clinic.	Postoperative Day 10

## Discussion

Penetrating tracheobronchial injuries, particularly those caused by gunshot wounds to the thoracic region, are critical surgical events with a low incidence (less than 1% of chest trauma) but high mortality rates [1,2,7]. The severity of the presented case was magnified not only by the primary tracheal damage (80% laceration) but also by the presence of an associated esophageal co-injury [3]. The combined compromise of both structures drastically increases the risk of fatal complications, most notably fulminant mediastinitis and the formation of a devastating TEF [8].

The successful management of this patient relied on the immediate securement of the airway and the early application of Damage Control Surgery (DCS) principles, which prioritize rapid hemorrhage and contamination control given the patient's hemodynamic instability and intraoperative circulatory arrest [9]. While median sternotomy is typically the preferred incision for superior and mid-thoracic tracheal exposure [4], it proved insufficient when cardiac arrest occurred. The immediate decision to perform an emergent left anterior thoracotomy, a rapid-access incision for resuscitation [5], was paramount. This allowed direct cardiac massage, restoration of spontaneous circulation, and adequate access to the posterior mediastinum, facilitating definitive repair and drainage of the esophageal contamination. This combined surgical approach (sternotomy plus thoracotomy) is an aggressive yet life-saving strategy in managing physiological collapse and the need for simultaneous access to multiple, severely injured structures [9].

For the definitive repair, a primary, single-stage reconstruction of both the trachea (end-to-end tracheoplasty) and the esophagus was chosen [10]. This approach is favored over delayed management, which is associated with increased morbidity [6]. A critical step in preventing recurrence and the formation of a TEF is the interposition of viable tissue between the suture lines [3,8]. In this case, the utilization of a pedicled intercostal muscle patch placed between the esophageal and tracheal repairs provided an essential, vascularized, and robust separation layer [3]. The patient’s rapid and favorable outcome, including extubation on postoperative Day 3 and confirmation via rigid or flexible bronchoscopy [11,12], validates the efficacy of prompt diagnosis and the rigorous, multilayered repair strategy.

This case serves as a vital teaching example: while standard surgical protocols exist [7], trauma surgeons must be prepared to deviate from customary surgical approaches [4] and employ combined, aggressive tactics (sternotomy plus emergency thoracotomy) when managing critical complications or profound hemodynamic instability. Reports like this are crucial for expanding the surgical literature on complex, acute penetrating trauma management, especially where the application of DCS principles dictates an unconventional surgical strategy.

## Conclusions

Thoracic tracheoesophageal injury following a gunshot wound represents a highly lethal surgical emergency that requires swift decision-making and definitive repair. The successful outcome in this case highlights the crucial role of an aggressive and multi-modal surgical strategy, particularly when initial stabilization is challenged by severe hemodynamic instability. The necessity of an emergent left anterior thoracotomy following an initial median sternotomy to manage an unexpected intraoperative cardiac arrest underscores that a dual surgical approach is a viable and life-saving strategy in severe penetrating chest trauma. This maneuver not only allowed for direct cardiac massage and the restoration of spontaneous circulation but also provided the necessary access to secure a complex esophageal injury.

The definitive management, characterized by a primary end-to-end tracheoplasty using interrupted monofilament absorbable sutures and a meticulous two-layer esophageal repair, demonstrates that single-stage reconstruction is feasible even in critical scenarios. Furthermore, the use of a pedicled intercostal muscle patch as a vascularized barrier proved essential in preventing TEF formation, a common and morbid complication. This case contributes to the surgical literature by demonstrating that preparedness to employ combined, non-standard surgical approaches is paramount when critical complications arise. Early recognition of airway disruption, multidisciplinary coordination, and surgical flexibility, transitioning from standard protocols to resuscitative maneuvers, remain the essential determinants of survival in complex, life-threatening airway trauma.
